# Optimal Periodic Cooperative Spectrum Sensing Based on Weight Fusion in Cognitive Radio Networks

**DOI:** 10.3390/s130405251

**Published:** 2013-04-19

**Authors:** Xin Liu, Min Jia, Xuemai Gu, Xuezhi Tan

**Affiliations:** 1 Communication Research Center, Harbin Institute of Technology, Harbin 150080, Heilongjiang, China; E-Mail: tanxz1957@hit.edu.cn; 2 College of Astronautics, Nanjing University of Aeronautics and Astronautics, Nanjing 210016, Jiangsu, China; 3 School of Electrical and Electronic Engineering, Nanyang Technological University, 637553, Singapore

**Keywords:** cognitive radio, energy sensing, cooperative spectrum sensing, sensing period, throughput

## Abstract

The performance of cooperative spectrum sensing in cognitive radio (CR) networks depends on the sensing mode, the sensing time and the number of cooperative users. In order to improve the sensing performance and reduce the interference to the primary user (PU), a periodic cooperative spectrum sensing model based on weight fusion is proposed in this paper. Moreover, the sensing period, the sensing time and the searching time are optimized, respectively. Firstly the sensing period is optimized to improve the spectrum utilization and reduce the interference, then the joint optimization algorithm of the local sensing time and the number of cooperative users, is proposed to obtain the optimal sensing time for improving the throughput of the cognitive radio user (CRU) during each period, and finally the water-filling principle is applied to optimize the searching time in order to make the CRU find an idle channel within the shortest time. The simulation results show that compared with the previous algorithms, the optimal sensing period can improve the spectrum utilization of the CRU and decrease the interference to the PU significantly, the optimal sensing time can make the CRU achieve the largest throughput, and the optimal searching time can make the CRU find an idle channel with the least time.

## 1. Introduction

With the rapid increase of communication demands, the spectrum layout based on the static spectrum allocation methodology has caused a shortage of spectrum resources [1]. Measurements by the Federal Communications Commission (FCC) have shown that 70% of the allocated spectrum in the US. has not been well utilized [2]. In order to improve the utilization of the finite spectrum sources, a new intelligent communication system named cognitive radio (CR) is proposed. CR, which is based on software radio, can reuse the radio spectrum that has been allocated to a primary user (PU) but is temporally unused [3]. Therefore, CR technique can improve the spectrum utilization greatly through operating on the idle channel.

Energy sensing which is independent of the prior information about PU, is used by cognitive radio user (CRU) frequently because of its simple and practicable implementation [4]. However, the performance of energy sensing can be degraded in the fading or shadow environment [5]. It has been proven that cooperative spectrum sensing outperforms single-user detection, which combines the detection results of multiple users [6]. In cooperative spectrum sensing, every collaborative CRU senses spectrum independently by energy sensing, and then sends its sensing information to a fusion centre that makes a final decision on the presence of PU through combining all the received sensing information [7].

Light-weight cooperative spectrum sensing based on hard decisions was proposed by Mishra in order to improve the detection probability in the given false alarm probability scenario [8]. By its predominant soft decision nature, an optimal linear operation framework for cooperative spectrum sensing based on weight fusion was proposed by Zhi in order to improve the sensing performance [9]. However, the false alarm probability which is related to the spectrum utilization of CRU was not considered in [8,9].

For improving the detection efficiency, a periodic spectrum detection model was proposed by Wang [10], which might decrease interference to the PU. A sensing-throughput tradeoff model was proposed in [11], which maximized the throughput of CRU by selecting an optimal sensing time. However in this model, CRU had to vacate the occupied channel and search for another idle channel so that its transmission could be continued when the presence of the PU was detected. The first problem studied in [11] was to minimize the search time while guaranteeing enough detection probability for CRU to find at least one idle channel. Once the average searching time was confirmed, the sensing time was then optimized in order to make CRU achieve maximal throughput. In [12], the sensing period was optimized for improving the idle spectrum access of CRU, however, the interference to PU was not considered by the authors. The proposed models of [10–12] were all based on single-user detection, and the cooperative spectrum sensing models based on sensing-throughput tradeoff were proposed in [13–15], which could improve the throughput of CRU on the premise of guaranteeing detection performance. However, the cooperative overhead generated by the models of [13–15] decreases the transmission time with the increase of the number of cooperative users.

In this paper, a new cooperative spectrum sensing model based on soft decision is proposed. At the same time, the sensing period, the sensing time, and the searching time are well considered, which are all optimized in order to improve the performance of the CRU observably, including increasing the spectrum utilization, decreasing the interference, improving the throughput and reducing the searching time. The fusion center combines the sensing information from cooperative users with the selected optimal weight factors in order to decrease false alarm probability and improve spectrum utilization. Firstly the sensing period is optimized for improving spectrum access and reducing interference to PU, then both the local sensing time and the number of cooperative users are jointly optimized in order to make CR achieve the maximal throughput during each period, and finally the water-filling principle is adopted to obtain the minimal searching time. The simulation results show that the proposed optimization scheme improves the sensing performance and decreases the interference to PU significantly.

The rest of this paper is organized as follows: Energy sensing model, cooperative sensing model, and primary user occupation model are described in Section 2, respectively, then sensing period, sensing time, and searching time are analyzed and optimized in Section 3, respectively, and the performance of the proposed optimization scheme is evaluated by the simulations in Section 4. Finally, the conclusions are drawn in Section 5.

## 2. Cooperative Spectrum Sensing

Common notation as summarized in Table 1 is used throughout this paper.

### 2.1. Energy Sensing

**Figure 1.**
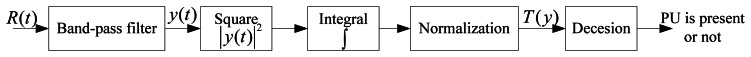
Energy sensing model.

Suppose that the centre frequency and bandwidth of the frequency band allocated to PU are *f_c_* and *W*, respectively, and the received signal is sampled at sampling frequency *f_s_* through the band-pass filter. The energy sensing model is shown in Figure 1, where the received signal *R*(*t*) is firstly passed through a band-pass filter with centre frequency *f_c_* and bandwidth *W* for getting the sampling signal in the frequency band of PU. The output of the filter *y*(*t*) is squared and integrated during the observed time *_T_* in order to obtain the energy of the received signal, then the energy statistic *T*(*y*) is obtained by normalizing the output of the integrator, and finally *T*(*y*) is compared with a threshold *λ* to decide whether PU is present or not.

The spectrum sensing problem can be seen as a binary hypothesis problem, which is given by:

(1) $$y\left(t\right)=\{\begin{array}{ll} u\left(t\right), & H_{0}\\ hs\left(t\right)+u\left(t\right), & H_{1} \end{array}\text{for}\;t=1,2,\ldots ,M$$

where *y*(*t*) is the sampled received signal, *s*(*t*) is the PU's signal with mean 0 and variance $\sigma_{s}^{2}$, *h* is the channel gain between PU and CR, *u*(*t*) is the Gaussian noise with mean 0 and variance $\sigma_{u}^{2}$, and *M* =*Tf_s_* is the number of samples. The statistic of energy sensing is obtained as follows:

(2) $$T\left(y\right)=\frac{1}{M}\sum_{t=1}^{M}{\left|y(t)\right|}^{2}$$

If *M* ≥ 100, according to the Centre Limit Theorem (CRT), *T*(*y*) approximates to obey the Gaussian distribution, whose mean and variance under *H*_0_ are respectively given by:

(3) $$\{\begin{array}{l} \text{E}\left(T\left(y\right)|H_{0}\right)=\sigma_{u}^{2}\\ \text{Var}\left(T\left(y\right)|H_{0}\right)=\frac{1}{M}\sigma_{u}^{4} \end{array}$$

By comparing *T*(*y*) with the threshold *λ*, the false alarm probability *P_f_* is obtained by:

(4) $$P_{f}=P_{r}\left(T\left(y\right)>\lambda |H_{0}\right)=Q\left(\left(\frac{\lambda }{\sigma_{u}^{2}}-1\right)\sqrt{Tf_{s}}\right)$$

where function $Q\left(x\right)=\frac{1}{\sqrt{2\pi }}\int_{x}^{\infty }\text{exp}\left(-\frac{x^{2}}{2}\right)dx$. According to Equations (1) and (2), the mean and variance of *T*(*y*) under *H*_1_ are respectively given by

(5) $$\{\begin{array}{l} \text{E}\left(T\left(y\right)|H_{1}\right)=\left(1+\gamma \right)\sigma_{u}^{2}\\ \text{Var}\left(T\left(y\right)|H_{1}\right)=\frac{1}{M}(1+2\gamma )\sigma_{u}^{4} \end{array}$$

where $\gamma =h^{2}\sigma_{s}^{2}/\sigma_{u}^{2}$ is the received signal noise rate (SNR) at CRU. Then the detection probability *P_d_* is given by:

(6) $$P_{d}=P_{r}\left(T\left(y\right)>\lambda |H_{1}\right)=Q\left(\left(\frac{\lambda }{\sigma_{u}^{2}}-\gamma -1\right)\sqrt{\frac{Tf_{s}}{2\gamma +1}}\right)$$

Hence, the miss detection probability is given by *P_m_* = 1− *P_d_*. On the other hand, by Equation (6), the threshold λ can also be related to the detection probability as follows:

(7) $$\lambda =\left(\sqrt{\frac{2\gamma +1}{Tf_{s}}}Q^{-1}\left(P_{d}\right)+\gamma +1\right)\sigma_{u}^{2}$$

By substituting Equation (7) into Equation (4), the false alarm probability is related to the detection probability as follows:

(8) $$P_{f}=Q\left(\sqrt{2\gamma +1}Q^{-1}\left(P_{d}\right)+\gamma \sqrt{Tf_{s}}\right)$$

while the detection probability is related to the false alarm probability as follows:

(9) $$P_{d}=Q\left(\frac{Q^{-1}\left(P_{f}\right)-\gamma \sqrt{Tf_{s}}}{\sqrt{2\gamma +1}}\right)$$

### 2.2. Cooperative Spectrum Sensing

Since if CRU is hidden by shadow or severe multipath fading, the sensing performance of single CRU is not accurate because of the received feeble power from PU, cooperative spectrum sensing is commonly used by CRU to solve hidden terminal problem [16].

**Figure 2.**
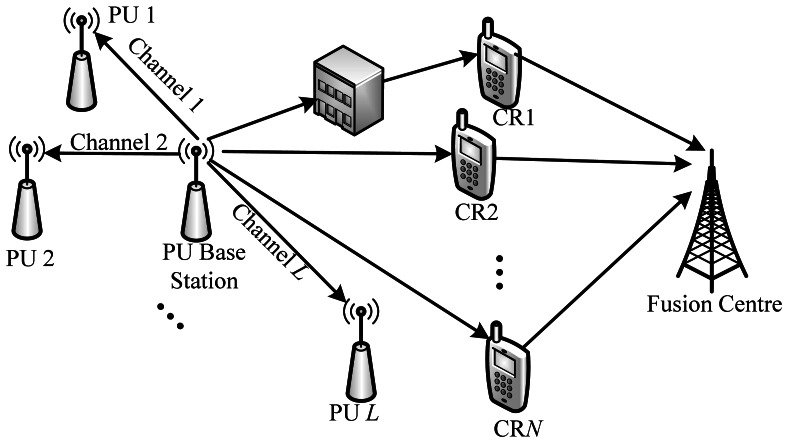
Cognitive radio networks.

As shown in Figure 2, we consider a CR network where there are *N* CRUs that act as the sensor nodes to detect the presence of PU cooperatively and *L* channels that can be used by CRU if PU is idle. In this figure, CRU1 is a hidden terminal, and CRU2–CRU*N* are collaborative terminals. These CRUs send their local observed information to a fusion center that functions as a base station, and then the fusion center combines all the received information to obtain a final decision on the presence of PU. With the cooperative help of CRU2–CRU*N*, the sensing performance of CRU1 can be improved greatly. Since PU may appear in the channel at any time, CRU needs to detect PU periodically in order to find its presence in time [17].

**Figure 3.**
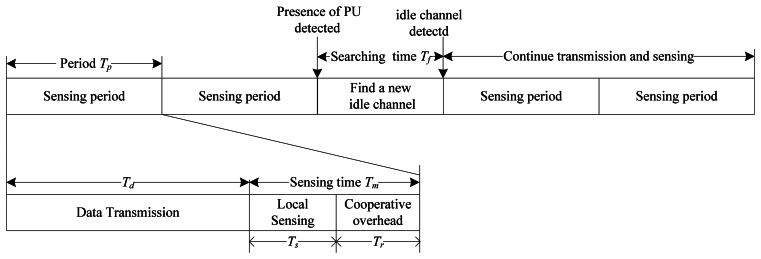
Periodic cooperative sensing model.

The periodic cooperative spectrum sensing model is shown in Figure 3. If an idle channel is detected currently, CRU can transmit and sense in this channel periodically. During each sensing period, after *T_d_* data transmission time, *T_m_* sensing time is needed to detect the presence of PU in order to avoid causing interference to PU. If the absence of PU is detected, CRU will repeat the process described above within the sensing period *T_p_*, however, if the presence of PU is detected during *T_m_*, CRU should vacate this channel at once, and search for a new idle channel from the left *L*-1 channels during the searching time *T_f_*. If another idle channel is found, CRU can switch to the channel and continue periodic transmission and sensing. Since CRUs detect PU by performing cooperative spectrum sensing, the cooperative overhead time *T_r_* is used for exchanging the signaling information. The transmission time *T_d_* is given by:

(10) $$T_{d}=T_{p}-T_{m}=T_{p}-T_{s}-T_{r}$$

Hence, a large *T_r_* may decrease *T_d_*, which induces the debasement on the throughput of CRU.

### 2.3. Primary User Occupation Model

Supposing that each channel is independent, the busy/idle state of the channel can be modeled as the Markov random process with ON/OFF type [18]. Therefore to the channel *j* for *j* ∈ [1, *L*] the persistent time of ON/OFF state obeys the exponential distribution with mean *u_j_*/*v_j_*, which is given by:

(11) $$\{\begin{array}{cc} f_{B_{j}}\left(t\right)=u_{j}e^{-u_{j}t}, & ON\\ f_{I_{j}}\left(t\right)=v_{j}e^{-v_{j}t}, & \text{OFF} \end{array}$$

where *u_j_* and *v_j_* are the transition rates from busy to idle and idle to busy, respectively. The busy and idle probabilities of each channel $P_{on}^{j}$ and $P_{\text{off}}^{j}$ for *j* = 1,2,…,*L* are respectively given as follows:

(12) $$\{\begin{array}{cc} P_{on}^{j} & =v_{j}/\left(u_{j}+v_{j}\right)\\ P_{\text{off}}^{j} & =u_{j}/\left(u_{j}+v_{j}\right) \end{array}$$

## 3. Optimal Cooperative Spectrum Sensing

### 3.1. Optimal Sensing Period

With the increase of sensing period *T_p_*, both the interference to PU and the loss of spectrum access may increase, while with the decrease of *T_p_*, the sensing time may increase because of frequent detection, and therefore it is important to choose an appropriate *T_p_* for CRU to achieve the maximal benefit. The sensing period of each channel is related with the activity of PU in this channel, and therefore the effect on *T_p_* caused by PU should be firstly analyzed.

Suppose that $T_{B}^{j}$ and $T_{I}^{j}$ denote the average persistent busy time and idle time of channel *j* during *T_p_* respectively, when the channel is ON at the initial time and OFF after *t*. If CRU detects the absence of PU falsely, it may use this channel and cause interference to PU during $T_{B}^{j}$, while if CRU detects the presence of PU falsely, CRU may lose the opportunity to access the idle channel during $T_{I}^{j}$. $T_{B}^{j}$ and $T_{I}^{j}$ are respectively given by:

(13) $$\{\begin{array}{c} T_{B}^{j}=P_{on}^{j}\int_{0}^{T_{p}}tf_{B_{j}}\left(t\right)dt=\frac{v_{j}}{u_{j}+v_{j}}\left(\frac{1}{u_{j}}\left(1-e^{-u_{j}T_{p}}\right)-T_{p}e^{-u_{j}T_{p}}\right)\\ T_{I}^{j}=P_{\text{off}}^{j}\int_{0}^{T_{p}}tf_{I_{j}}\left(t\right)dt=\frac{u_{j}}{u_{j}+v_{j}}\left(\frac{1}{v_{j}}\left(1-e^{-v_{j}T_{p}}\right)-T_{p}e^{-v_{j}T_{p}}\right) \end{array}$$

Since the optimization of sensing period is the first step to optimize cooperative spectrum sensing, false alarm probability *Q_f_* and detection probability *Q_d_* cannot be obtained accurately. Hence, according to the characters of CR in IEEE 802.22, the constraints of *Q_f_* and *Q_d_* need to be set in order to guarantee spectrum utilization and avoid interfering PU.

CRU should have a lower false alarm probability in order to improve its spectrum utilization, and therefore *P_f_* must be below a certain value, which may keep CRU owning sufficient spectrum resources for transmitting data. While CRU should have a high detection probability in order to avoid causing great interference to PU, and thus *P_d_* must be above a certain value, which may make CRU give a more accurate detection on the presence of PU. Commonly, the constraints can be given by:

(14) $$\{\begin{array}{c} P_{f}\leq {\bar{P}}_{f}\leq 0.5\\ P_{d}\geq {\bar{P}}_{d}\geq 0.5 \end{array}$$

where *P̅*_f_ and *P̅*_d_ are the upper limit of false alarm probability and the lower limit of detection probability respectively. In the optimization of sensing period, we use the constraints *P̅_f_* and *P̅_d_* instead of the actual probabilities *P_f_* and *P_d_*.

**Figure 4.**
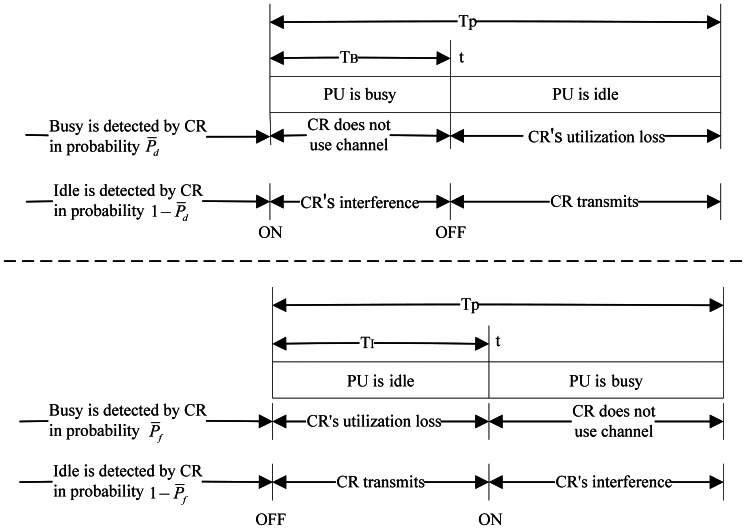
Interference and loss of spectrum access during one period.

Figure 4 shows the interference to PU and the loss of spectrum access during one period. If PU is factually present at the initial time and the busyness of the channel was detected accurately during the previous period, CRU will avoid using this channel during this period and therefore have $T_{p}-T_{B}^{j}$ loss of spectrum access in probability *P̅_d_*. While if the idleness of the channel is detected falsely during the previous period, CRU will occupy this channel and cause $T_{B}^{j}$ interference to PU in probability 1−*P̅_d_*. Similarly, if PU is factually absent at the initial time, there will be $T_{I}^{j}$ loss of spectrum access in probability *P̅*_d_ and $T_{p}-T_{I}^{j}$ interference to PU in probability 1−*P̅_f_* Hence, the average loss time of spectrum access and the average interference time are respectively given as follows:

(15) $$\{\begin{array}{l} T_{\text{loss}}=\frac{1}{L}\sum_{j=1}^{L}\left({\bar{P}}_{d}\left(T_{p}-T_{B}^{j}\right)+{\bar{P}}_{f}T_{I}^{j}\right)\\ T_{\text{inf}}=\frac{1}{L}\sum_{j=1}^{L}\left(\left(1-{\bar{P}}_{d}\right)T_{B}^{j}+\left(1-{\bar{P}}_{f}\right)(T_{p}-T_{I}^{j})\right) \end{array}$$

CRU must sense PU during each period, and therefore when CRU is sensing a channel, it has to stop transmitting in this channel, and cause the loss of spectrum access that is called sensing overhead and denoted by *TS_loss_*. Supposing that *T_m,max_* is the maximum of sensing time during each period, *TS_loss_* is given by:

(16) $${\text{TS}}_{\text{loss}}=\frac{1}{L}\sum_{j=1}^{L}T_{m,\text{max}}\left(\left(1-{\bar{P}}_{d}\right)P_{on}^{j}+(1-{\bar{P}}_{f})P_{\text{off}}^{j}\right)$$

In order to improve spectrum utilization and decrease interference to PU, loss time, interference time and sensing overhead are considered synthetically, and the minimal sensing loss can be achieved through optimizing the sensing period, whose optimization problem is defined as follows:

(17) $$\begin{array}{l} T_{p}^{*}=\underset{T_{p}}{\text{argmin}}\left(Q_{\text{loss}}=\frac{\eta_{1}\left(T_{\text{loss}}+TS_{\text{loss}}\right)+\eta_{2}T_{\text{inf}}}{T_{p}}\right)\\ s.t.T_{p}\geq T_{m,\text{max}} \end{array}$$

where *Q_loss_* denotes the total sensing loss probability, and *η*_1_ and *η*_2_ are the weight factors configured by the CR network. Since η_1_(*T_loss_* + *TS_loss_*) and η_2_*T_inf_* denote the loss of spectrum access and the interference to PU respectively, the selection of *η*_1_ and *η*_2_ should satisfy the needs of CRU. Large *η*_1_ can improve the spectrum utilization of CR, while large *η*_2_ can decrease the interference to PU.

To describe the effect on the sensing performance of selecting *T_p_*, two measurements that the probability of spectrum utilization *ζ_use_* and the probability of interference *ζ_inf_* are defined as follows:

(18) $$\{\begin{array}{cc} \zeta_{\text{use}} & =\frac{T_{p}-T_{\text{loss}}-TS_{\text{loss}}}{T_{p}\left(\left(1-{\bar{P}}_{d}\right){\bar{P}}_{on}+(1-{\bar{P}}_{f}){\bar{P}}_{\text{off}}\right)}\\ \zeta_{\text{inf}} & =\frac{T_{\text{inf}}}{T_{p}\left(\left(1-{\bar{P}}_{d}\right){\bar{P}}_{on}+(1-{\bar{P}}_{f}){\bar{P}}_{\text{off}}\right)} \end{array}$$

where ${\bar{P}}_{on}=\frac{1}{L}\sum_{j=1}^{L}P_{on}^{j}$ and ${\bar{P}}_{\text{off}}=\frac{1}{L}\sum_{j=1}^{L}P_{\text{off}}^{j}$.

**Figure 5.**
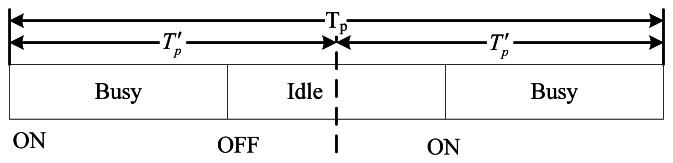
Sensing period including two state transitions.

In the description above, we suppose that only one ON/OFF or OFF/ON state transition happens during one period, however, if larger sensing period *T_p_* is chosen, there may be multiple state transitions during one period. In order to solve the problem, we need to divide *T_p_* into multiple sub-periods. For example, as shown in Figure 5, PU undergoes both ON/OFF and OFF/ON state transitions during *T_p_*, and after dividing *T_p_* into two sub-periods $T_{p}^{'}$, PU undergoes only one ON/OFF or OFF/ON state transition during $T_{p}^{'}$. Hence, we can use the optimization of sensing period mentioned above to obtain the optimal $T_{p}^{'}$. Choosing an appropriate sensing period is very important, if sensing period is too large or too small, spectrum sensing will not find the appearance of PU in time, and therefore CRU may cause great interference to PU.

### 3.2. Optimal Sensing Time

As shown in Figure 3, the sensing time *T_m_* includes local sensing time *T_s_* and cooperative overhead *T_r_*. Each CRU senses PU for obtaining its local detection result by energy sensing independently during the local sensing time, and then these cooperative CRUs send their sensing information to the fusion centre one by one instead of synchronous transmission in order to avoid causing larger channel consumption and transmission collision to the CR network. Since the cooperative overhead time *T_r_* is proportional to the number of the CRUs participating in cooperative spectrum sensing denoted by *n* for 1≤ *n* ≤ *N*, we have *T_r_*=*nξ* where *ξ* denotes the time used for transmitting sensing information by each CRU. After the fusion centre receives the sensing information from all the collaborative CRUs, it may combine these information by the weight vector ***ω*** = [*ω*_1_, *ω*_2_,…,*ω_n_*] where ‖***ω***‖ = 1. Hence, the fusion statistic is given by:

(19) $$Z=\sum_{i=1}^{n}\omega_{i}g_{i}T(y_{i})$$

where *g_i_* for *i* =1,2,..,*n* is the channel gain from CRU*i* to the fusion centre. According to Equations (3), (5) and (19), the means and variances of *Z* under *H*_0_ and *H*_1_ are respectively given by:

(20) $$\{\begin{array}{l} \text{E}\left\{Z|H_{0}\right\}=\sum_{i=1}^{n}\omega_{i}g_{i}\text{E}\left\{T(y_{i})|H_{0}\right\}=\sum_{i=1}^{n}\omega_{i}g_{i}\sigma_{u}^{2}\\ \text{E}\left\{Z|H_{1}\right\}=\sum_{i=1}^{n}\omega_{i}g_{i}\text{E}\left\{T(y_{i})|H_{1}\right\}=\sum_{i=1}^{n}\omega_{i}g_{i}\left(1+\gamma_{i}\right)\sigma_{u}^{2}\\ \text{Var}\left\{Z|H_{0}\right\}=\sum_{i=1}^{n}\omega_{i}^{2}g_{i}^{2}\text{Var}\left\{T(y_{i})|H_{0}\right\}=\frac{1}{M}\sum_{i=1}^{n}\omega_{i}^{2}g_{i}^{2}\sigma_{u}^{4}\\ \text{Var}\left\{Z|H_{1}\right\}=\sum_{i=1}^{n}\omega_{i}^{2}g_{i}^{2}\text{Var}\left\{T(y_{i})|H_{1}\right\}=\frac{1}{M}\sum_{i=1}^{n}\omega_{i}^{2}g_{i}^{2}\left(1+2\gamma_{i}\right)\sigma_{u}^{4} \end{array}$$

where *γ_i_* is the received SNR of CRU*i*. Like Equations (6) and (8), the cooperative false alarm probability *Q_f_* and detection probability *Q_d_* are obtained as follows:

(21) $$\{\begin{array}{l} Q_{f}=Q\left(\frac{\lambda -\sum_{i=1}^{n}\omega_{i}g_{i}\sigma_{u}^{2}}{\sqrt{\frac{1}{T_{s}f_{s}}\sum_{i=1}^{n}\omega_{i}^{2}g_{i}^{2}\sigma_{u}^{4}}}\right)\\ Q_{d}=Q\left(\frac{\lambda -\sum_{i=1}^{n}\omega_{i}g_{i}\left(1+\gamma_{i}\right)\sigma_{u}^{2}}{\sqrt{\frac{1}{T_{s}f_{s}}\sum_{i=1}^{n}\omega_{i}^{2}g_{i}^{2}\left(1+2\gamma_{i}\right)\sigma_{u}^{4}}}\right) \end{array}$$

According to Equations (7) and (21), for a target detection probability *P̅_d_*, the cooperative false alarm probability is related to the target detection probability as follows

(22) $$Q_{f}=Q\left(\frac{Q^{-1}\left({\bar{P}}_{d}\right)\sqrt{\frac{1}{T_{s}f_{s}}\sum_{i=1}^{n}\omega_{i}^{2}g_{i}^{2}\left(1+2\gamma_{i}\right)}+\sum_{i=1}^{n}\omega_{i}g_{i}\gamma_{i}}{\sqrt{\frac{1}{T_{s}f_{s}}\sum_{i=1}^{n}\omega_{i}^{2}g_{i}^{2}}}\right)$$

By supposing that γ̅ is the average received SNR of all the CRUs, we can obtain that

(23) $$Q_{f}\approx Q\left(Q^{-1}\left({\bar{P}}_{d}\right)\sqrt{1+2\bar{\gamma }}+\frac{\sum_{i=1}^{n}\omega_{i}g_{i}\gamma_{i}}{\sqrt{\frac{1}{T_{s}f_{s}}\sum_{i=1}^{n}\omega_{i}^{2}g_{i}^{2}}}\right)$$

where the optimal weight vector ***ω*** is obtained by minimizing *Q_f_*. Since *Q*(*x*) is a monotone decreasing function, according to Equation (23), the optimization of *Q_f_* can be converted as follows:

(24) $$\begin{array}{l} \omega^{*}=\underset{\omega }{\text{argmax}}\left(f\left(\omega \right)=T_{s}f_{s}\frac{{\left(\sum_{i=1}^{n}\omega_{i}g_{i}\gamma_{i}\right)}^{2}}{\sum_{i=1}^{n}\omega_{i}^{2}g_{i}^{2}}\right)\\ s.t.\omega =1 \end{array}$$

To solve Equation (24), the linear transformation is operated as follows

(25) $$\beta =\Sigma^{1/2}\omega$$

where vector $\Sigma =\text{diag}\left[g_{1}^{2},g_{2}^{2},\ldots ,g_{n}^{2}\right]$ where diag[***x***] denotes the diagonal matrix by using *x* as the diagonal elements. By substituting Equations (25) into Equation (24), according to the Rayleigh Ritz Inequality [19], we have:

(26) $$\begin{array}{ll} f\left(\omega \right) & =T_{s}f_{s}\frac{\beta^{T}\Sigma^{-T/2}\Psi \Psi^{T}\Sigma^{-1/2}\beta }{\beta^{T}\beta }\\ & \leq \lambda_{\text{max}}T_{s}f_{s}\left(\Sigma^{-T/2}\Psi \Psi^{T}\Sigma^{-1/2}\right)\\ & =T_{s}f_{s}{\left\|\Sigma^{-T/2}\Psi \right\|}^{2} \end{array}$$

where ***ψ*** = [g_1_γ_1_, g_2_γ_2_, …, g_2_γ_2_], *λ*_max_ is the maximal eigenvalue of the positive semi-definite matrix ***Σ***^−T/2^***ψψ****^T^* ***Σ***^−1/2^ [9], and the equality to maximize *f*(***ω***) is given by:

(27) $$\beta =\Sigma^{-T/2}\Psi$$

By substituting Equation (27) into Equation (25), we have:

(28) $$\omega =\Sigma^{-1/2}\Sigma^{-T/2}\Psi =\Sigma^{-1}\Psi$$

With the restriction condition ‖***ω***‖=1, the optimal weight factors is obtained as follows:

(29) $$\begin{array}{cc} \omega_{i}^{*}=\frac{\omega_{i}}{\left\|\omega \right\|}=\frac{\gamma_{i}}{g_{i}}/\sqrt{\sum_{i=1}^{n}\frac{\gamma_{i}^{2}}{g_{i}^{2}}}, & i=1,2,\ldots ,n \end{array}$$

by substituting Equation (29) into Equation (23), the minimal false alarm probability is given by:

(30) $$Q_{f,\text{min}}=Q\left(Q^{-1}\left({\bar{P}}_{d}\right)\sqrt{1+2\bar{\gamma }}+\sqrt{T_{s}f_{s}\sum_{i=1}^{n}\gamma_{i}^{2}}\right)$$

By comparing Equation (30) with Equation (8), it is seen evidently that with *n* ≥ 1, *Q_f,min_* < *P_f_* which decreases with the increase of *n*. Suppose that *C*_0_ and *C*_1_ are the throughput of CRU operating in the idle channel and the busy channel respectively. For example, if there is only point-to-point transmission in the CR network, by assuming that the signals of PU and CRU are independent of each other, we can obtain *C*_0_ and *C*_1_ as follows:

(31) $$\{\begin{array}{l} C_{0}=W{\text{log}}_{2}\left(1+\frac{P_{R}}{\sigma_{u}^{2}}\right)\\ C_{1}=W{\text{log}}_{2}\left(1+\frac{P_{R}}{\sigma_{s}^{2}+\sigma_{u}^{2}}\right) \end{array}$$

where *P_R_* is the transmission power of CRU. Obviously, we have *C*_0_ > *C*_1_. Note that if the signals are not independent, the above formula for *C*_0_ and *C*_1_ can be treated as the upper bound of the achievable rate when PU is active or not.

There are following two scenarios where CRU can operate in the frequency band of PU [19].

(1) If PU is absent and CRU detects idleness of the channel exactly, the achievable throughput of CRU is (1 − *Q_f_*)*C*_0_*T_d_* / *T_p_*;
(2) If PU is present but it is not detected by CRU, the achievable throughput of CRU is (1 − *Q_d_*)*C*_1_*T_d_* / *T_p_*.

Hence, the average throughput of CRU related to *T_s_* and *n* is given as follows:

(32) $$R\left(T_{s},n\right)=\frac{T_{p}-T_{s}-T_{r}}{T_{p}}\left(P_{\text{off}}C_{0}\left(1-Q_{f}\right)+P_{on}C_{1}\left(1-Q_{d}\right)\right)$$

Since the transmission time of CRU is *T_p_*−*T_s_*−*T_r_* and the probability of interfering PU is *P_on_*(1 − *Q_d_*), the average interfering time is given by:

(33) $$T_{I}=P_{on}\left(1-Q_{d}\right)\left(T_{p}-T_{s}-T_{r}\right)$$

Commonly, it is required that the average interfering time of CRU is less than a certain value, that is *T_I_* ≤ *μ*. The goal of optimizing sensing time is to identify the optimal local sensing time *T_s_* and number of cooperative CRUs *n* during each sensing period, so that the achievable throughput of the CR network is maximal while PU is protected sufficiently. Mathematically, the optimization problem is proposed as follows:

(34) $$\begin{array}{ll} \text{max} & R\left(T_{s},n\right)\\ s.t. & Q_{d}\geq {\bar{P}}_{d}\\ & Q_{f}\leq {\bar{P}}_{f}\\ & T_{I}\leq µ\\ & T_{s}+n\xi \leq T_{m,\text{max}}\\ & 1\leq n\leq N \end{array}$$

According to Equation (22), with the given *T_s_* and *n*, *Q_f_* may increase with the improvement of *Q_d_*, and according to Equation (32), both the increase of *Q_f_* and *Q_d_* may lead to the decrease of the average throughput *R*(*T_s_*, *n*). Therefore in order to maximize *R*(*T_s_*, *n*), *Q_d_* and *Q_f_* should both fall to their lower bounds, that is *Q_d_* = *P̅d* and *Q_f_* = *Q_f,min_* (as shown in Equation (30)). The optimal solution to Equation (34) is also achieved when the equality *Q_d_* = *P̅d* is satisfied, and supposing that *P̅d* ≈ 1, the optimization problem of Equation (34) is rewritten as follows:

(35) $$\begin{array}{l} \left[T_{s}^{*},n^{*}\right]=\underset{T_{s},n}{\text{argmax}}\left(\Phi \left(T_{s},n\right)=\frac{T_{p}-T_{s}-n\xi }{T_{p}}\left(1-Q\left(\alpha +\sqrt{T_{s}f_{s}\sum_{i=1}^{n}\gamma_{i}^{2}}\right)\right)\right)\\ \begin{array}{ll} s.t. & \varphi \leq T_{s}+n\xi \leq T_{m,\text{max}}\\ & 1\leq n\leq N \end{array} \end{array}$$

where *φ*=*T_p_*−μ/(*P_on_*(1 − *P̅_d_*)) and $\alpha =Q^{-1}\left({\bar{P}}_{d}\right)\sqrt{1+2\bar{\gamma }}$. From Equation (35), we can see that the optimized objective is a function of *T_s_* and *n*. If *n* is given, the sub-optimization of *T_s_* is firstly considered as follows:

**Proposition 1:** If *n* is given, there is an optimal *T_s_* ∈ [0,*T_p_*−*nξ*] that maximizes *Φ*(*T_s_*,*n*).
**Proof:** see Appendix.

According to the first constraint of Equation (35), we have *φ* −*nξ* ≤ *T_s_* ≤ *T_m,max_* − *nξ*. By Proposition 1, if ▽*Φ*(*T_m,max_* − *nξ*, *n*) > 0, the optimal $T_{s}^{*}=T_{m,\text{max}}-n\xi$, else if ▽*Φ*(*φ*− *nξ*, *n*) < 0, the optimal $T_{s}^{*}=\varphi -n\xi$, and otherwise $T_{s}^{*}$ is the maximal point of *Φ*(*T_s_*,*n*). With any given *n*, the iterative method to find an optimal $T_{s}^{*}$ is defined by the Algorithm 1.


**Algorithm 1.** With any given *n*, find an optimal
$T_{s}^{*}$ that makes *Φ*(*T_s_*,*n*)achieve maximum
Given *n* and *ε* (error precision
$T_{s}^{*}$), initialize *τ_min_* = *φ* − *nξ* and *τ_max_* = *T_m,max_* −*nξ*.If sign(▽*Φ*(*τ_min_*,*n*))==sign(▽*Φ*(*T_p_*,*n*)), let 
$T_{s}^{*}=\tau_{\text{min}}$;else if sign(▽*Φ*(*τ_max_*,*n*))== sign(▽*Φ*(0, *n*)), let 
$T_{s}^{*}=\tau_{\text{max}}$; else go to step 3.Let *τ*= (*τ_min_* +*τ_max_*)/2.If sign(▽*Φ*(*τ*,*n*))==sign(▽*Φ*(*τ_min_*,*n*)), let *τ_min_*=*τ*, and otherwise let *τ_max_* =*τ*.Repeat step 3∼4 until | *τ_max_*− *τ_min_*| ≤ *ε*.Let 
$T_{s}^{*}=\left(\tau_{\text{min}}+\tau_{\text{max}}\right)/2$.


The second sub-optimization problem is that with a given *T_s_*, how to find an optimal *n* that maximizes *Φ*(*T_s_*, *n*). Since *n* is an integer, it is not computationally expensive to search through *n* from 1 to *N*. The joint optimization algorithm of *T_s_* and *n* is shown in Algorithm 2.


**Algorithm 2.** Joint optimization algorithm of *T_s_* and *n*.
Array the *N* CRUs as 1,2,…,*N* in descending order of their SNRs.Initialize that 
$T_{s}^{\left(0\right)}$ equals to any value within (0, *T_m,max_*], *n*^(0)^=1 and *j* = 0.Given 
$T_{s}^{\left(j\right)}$, enumerate *n* from 1 to *N* and calculate corresponding
$\Phi \left(T_{s}^{\left(j\right)},n\right)$ with the first *n* CRUs.Find n* that maximizes 
$\Phi \left(T_{s}^{\left(j\right)},n\right)$ for *n*=1,2,…,*N*, and let *n*^(^*^j^*^+1)^ =n*.Given *n*^(^*^j^*^+1)^, calculate the optimal 
$T_{s}^{*}$ by Algorithm 1 and let 
$T_{s}^{\left(j+1\right)}=T_{s}^{*}$.Let *j* = *j*+1.Repeat step 3∼6 until 
$\left|T_{s}^{\left(j\right)}-T_{s}^{\left(j-1\right)}\right|<ɛ$ && *n*^(^*^j^*^)^== *n*^(^*^j^*−^1)^.Let 
$T_{s}^{*}=T_{s}^{\left(j\right)}$ and n*= *n*^(^*^j^*^)^.


The optimal sensing time $T_{m}^{*}$ can be given as follows:

(36) $$T_{m}^{*}=T_{s}^{*}+n^{*}\xi$$

### 3.3. Optimal Searching Time

**Figure 6.**
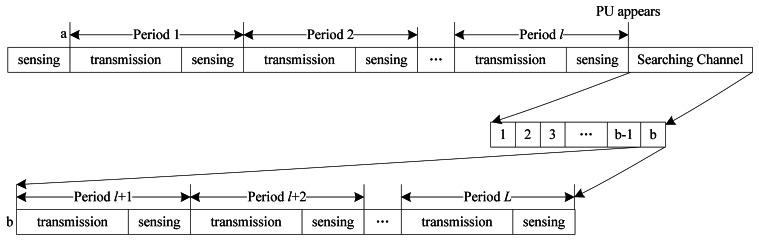
Processes of sensing spectrum and searching channel.

After several transmission periods, CRU may detect the appearance of the PU, and therefore CRU has to stop transmission and search for a new idle channel. Once CRU finds the idle channel, it will stop searching to continue transmission in the new channel. As shown in Figure 6, CRU firstly finds an idle channel *a* through spectrum sensing, and then performs transmitting and sensing in channel *a* periodically. If CRU detects the appearance of PU during the sensing time in period *l*, in order to avoid interfering PU, CRU has to search for another idle channel. CRU will detect the spare channels one by one until a new idle channel *b* is found, and then continue transmitting and sensing in channel *b* periodically. Therefore the optimization of searching time is related with the sensing time and idle probability of single channel.

The current research on searching time such as [11] focuses on the selection of searching type, however, the research assumes that the sensing time of single channel is same, which is based on single-user detection. In [11], the author minimized the average searching time through optimizing the same sensing time of single channel, and this optimization problem is defined as follows:

(37) $$\underset{T_{u}}{\min}\left(T_{f}=\frac{T_{u}}{\left(1-P_{f}\left(T_{u}\right)\right)P_{\text{off}}}\right)$$

where *T_u_* is the sensing time of single channel, and *P_f_*(*T_u_*) is the false alarm probability of single channel with sensing time *T_u_*.

Since the idle state of each channel is distinct, the selection of sensing time for single channel should also be different, and it is necessary to use a weight factor to allocate the sensing time for each channel. In this paper, the average searching time can achieve minimum through optimizing the weighed sensing time of single channel including local sensing time and cooperative overhead. Here continuous searching type that the channels are detected by CRU in turn is adopted, that is, if the busyness of the current searching channel is detected, CRU will select the next channel to continue detecting. Through multiplying the local sensing time by a weight factor decided by the idle state of single channel, the searching time $T_{u}^{j}$ of channel *j* is obtained as follows:

(38) $$\begin{array}{ll} T_{u}^{j}=T_{s}^{\prime j}+T_{r}^{\prime }=w_{j}{\bar{T}}_{s}^{\prime }+n^{\prime }\xi , & j=1,2,\ldots ,L \end{array}$$

where $T_{s}^{'j}$ and *w_j_* are the local sensing time and weight factor of channel *j* respectively, and $T_{s}^{'j}$, $T_{r}^{'}$ and n′ are the average sensing time, the cooperative overhead and the number of cooperative users in the searching process respectively. Since the total local sensing time of all the channels is $L{\bar{T}}_{s}^{'}$, we have $\sum_{j=1}^{L}w_{j}=L$. According to Equation (12), the busy probability of channel *j* detected by CRU is given by:

(39) $$Q_{b}^{j}=\left(1-P_{\text{off}}^{j}\right)Q_{d}^{j}+P_{\text{off}}^{j}Q_{f}^{j}$$

where $Q_{d}^{j}$ and $Q_{f}^{j}$ are the cooperative detection probability and false alarm probability of channel *j* respectively.

Since CRU can quickly find an idle channel with lower busy probability, the average searching time may degrade with the decrease of $Q_{b}^{j}$. According to Equations (30) and (39), the minimal $Q_{b,\text{min}}^{j}$ used for minimizing the average searching time *T_f_* is given if both of $Q_{d}^{j}$ and $Q_{f}^{j}$ reach their lower bounds. We have known that $Q_{d}^{j}\geq {\bar{P}}_{d}$ and $Q_{f}^{j}\geq Q_{f,\text{min}}^{j}$, and it is obtained that:

(40) $$Q_{b,\text{min}}^{j}=\left(1-P_{\text{off}}^{j}\right){\bar{P}}_{d}+P_{\text{off}}^{j}Q_{f,\text{min}}^{j}$$

According to Equations (30), (38), (39) and (40), $Q_{b,\text{min}}^{j}$ are related with the parameters *w_j_*, ${\bar{T}}_{s}^{'}$ and n′, and therefore the searching time is obtained as follows:

(41) $$\begin{array}{ll} T_{f}\left(w,{\bar{T}}_{s}^{'},n^{'}\right) & =T_{u}^{1}\left(1-Q_{b,\text{min}}^{1}\right)+\left(T_{u}^{1}+T_{u}^{2}\right)Q_{b,\text{min}}^{1}\left(1-Q_{b,\text{min}}^{2}\right)+\ldots +\left(T_{u}^{1}+T_{u}^{2}+\ldots +T_{u}^{L}\right)Q_{b,\text{min}}^{1}Q_{b,\text{min}}^{2}\ldots Q_{b,\text{min}}^{L-1}\left(1-Q_{b,\text{min}}^{L}\right)\\ & =\sum_{j=1}^{L}\left(\left(1-Q_{b,\text{min}}^{j}\right)\prod_{m=0}^{j-1}\left(Q_{b,\text{min}}^{m}\right)\sum_{k=1}^{j}T_{u}^{k}\right)\\ & =\sum_{j=1}^{L}\left(\left(1-Q_{b,\text{min}}^{j}\left(w_{j},{\bar{T}}_{s}^{'},n^{'}\right)\right)\prod_{m=0}^{j-1}\left(Q_{b,\text{min}}^{m}\left(w_{m},{\bar{T}}_{s}^{'},n^{'}\right)\right)\sum_{k=1}^{j}\left(w_{k}{\bar{T}}_{s}^{'}+n^{'}\xi \right)\right) \end{array}$$

where $T_{f}\left(w,{\bar{T}}_{s}^{'},n^{'}\right)$ and $Q_{b,\text{min}}^{j}\left(w_{j},{\bar{T}}_{s}^{'},n^{'}\right)$ denote the functions about the parameters ***w***, *w_j_*, ${\bar{T}}_{s}^{'}$ and n′, and the weight vector ***w***=[*w*_1_, *w*_2_,…,*w_L_*]. The optimization problem of the average searching time *T_f_* is given as follows:

(42) $$\begin{array}{l} \left[w^{*},{\bar{T}}_{s}^{'*},n^{'*}\right]=\underset{w,{\bar{T}}_{s}^{'},n^{'}}{\text{argmin}}T_{f}\left(w,{\bar{T}}_{s}^{'},n^{'}\right)\\ \begin{array}{ll} s.t. & {\bar{T}}_{s}^{'}+n^{'}\xi \leq T_{m,\text{max}}\\ & \sum_{j=1}^{L}w_{j}=L\\ & 1\leq n^{'}\leq N \end{array} \end{array}$$

The weight vector ***w*** is related with the idle probability *P^j^_off_* for *j* = 1,2,…,*L* and ***w*** is commonly selected as follows:

(43) $$\begin{array}{cc} w_{j}=L\frac{P_{\text{off}}^{j}}{\sum_{j=1}^{L}P_{\text{off}}^{j}}, & j=1,2,\ldots ,L \end{array}$$

which implies that the channel with large idle probability should be prior detected. However, this scheme makes CRU detect all the channels regardless of the inapplicable channel with lower idle probability, and therefore we seek to make the weight factors of these inapplicable channels zero in order to avoid detecting them for saving time. To solve the problem, the selection of ***w*** based on water-filling principle is proposed in Algorithm 3.

In the Algorithm 3, the channel with idle probability lower than water-filling threshold ϑ, which is frequently use by PU, will not be detected by CRU in order to save searching time. By the algorithm, the channel with higher idle probability may have a larger weight factor, while the channel with lower idle probability may have a smaller weight factor, and therefore the searching time can be allocated to each channel reasonably. Once ***w*** is obtained, the optimal ${\bar{T}}_{s}^{'}$ and n′ can be similarly determined by Algorithm 1 and Algorithm 2.


**Algorithm 3.** Selection of ***w*** based on water-filling principle.

(1)Array the *L* channels as an aggregation ***A*** = {1,2,…,*L*} in descending order of their idle probabilities $P_{\text{off}}^{j}$ for *j* = 1,2,…,*L*(2)Initialize water-filling threshold ϑ=0 and the number of the elements in ***A** size* (***A***) = *L*.(3)Calculate water-filling capacity $C=L+\sum_{j=1}^{L}{\left(P_{\text{off}}^{j}\right)}^{-1}$.(4)Update water-filling threshold as ϑ=*C*/ *size*(***A***).(5)If $\exists \vartheta -{\left(P_{\text{off}}^{j}\right)}^{-1}\leq 0$ for *j* ∈ ***A***, let $C=C-{\left(P_{\text{off}}^{j}\right)}^{-1}$ and ***A***=***A***−{*j*}; go to step 6.(6)Repeat step 4∼5 until $P_{\text{off}}^{j}\geq 1/\vartheta$, ∀ *j* ∈ ***A***.(7)Calculate the final water-filling threshold $\vartheta =\left(L+\sum_{j\in A}{\left(P_{\text{off}}^{j}\right)}^{-1}\right)/\text{size}\left(A\right)$, and the weight factors $w_{j}=\vartheta -{\left(P_{\text{off}}^{j}\right)}^{-1}$ for *j* ∈ ***A*** and *w_j_*=0 for *j* ∉ ***A***. Algorithm 3 ends.


## 4. Simulation

Suppose that the sampling frequency *f_s_* = 1 kHz, the upper limit of false alarm probability *P̅_f_*=0.4, the lower limit of detection probability *P̅_d_*=0.9, the number of CRUs *N* = 10, the number of available channels *L* = 10, the average received SNR γ̅ = −10∼0dB, the transition rates *u*, *v* =0∼0.1, the maximal sensing time *T_m,max_* = 1 s, the maximal interfering time *μ* = 0.4 s, the time of transmitting sensing information *ξ*=0.05 s, and the tolerant accuracy *ε* = 10^−5^.

**Figure 7.**
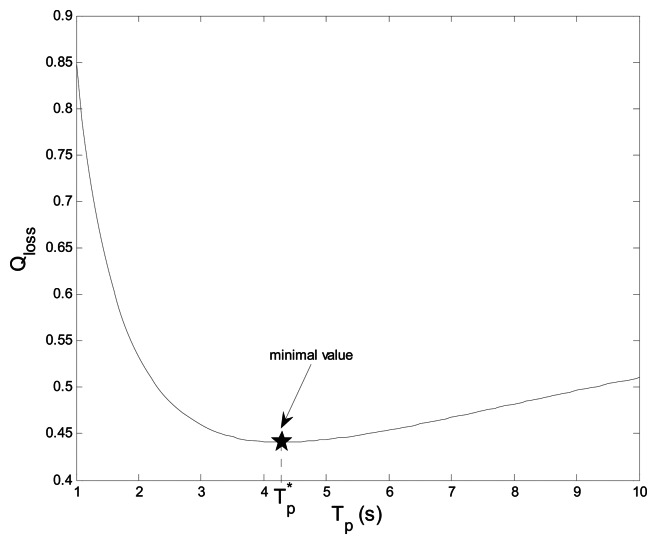
Total sensing loss probability *vs.* sensing period.

Figure 7 shows the total sensing loss probability *Q_loss_ vs.* sensing period *T_p_* with *η*_1_= *η*_2_ =1. There is an optimal T_p_* = 4.3 s that minimizes *Q_loss_*. When *T_m_*_,ma_*_x_* ≤ *T_p_* ≤ T_p_*, the higher sensing frequency leads to the increase of sensing time that may reduce the opportunity of spectrum access, and therefore *Q_loss_* increases. When *T_p_* >T_p_*, although the sensing frequency decreases, CRU can't detect the absence or presence of PU in time during larger *T_p_*, and therefore the increase of the loss of spectrum access and the interference to PU also leads to the increase of *Q_loss_*.

**Figure 8.**
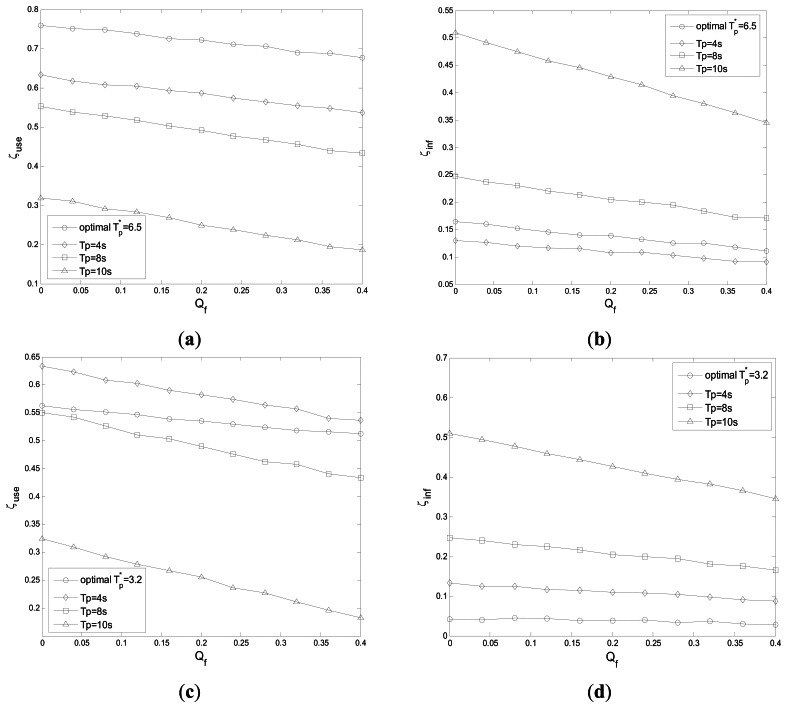
(**a**) Probability of spectrum utilization *vs.* cooperative false alarm probability (*η*_1_=1 and *η*_2_=0.1). (**b**) Probability of interference *vs.* cooperative false alarm probability (*η*_1_=1 and *η*_2_=0.1). (**c**) Probability of spectrum utilization *vs.* cooperative false alarm probability (*η*_1_=0.1and *η*_2_=1). (**d**) Probability of interference *vs.* cooperative false alarm probability (*η*_1_=0.1and *η*_2_=1).

Figure 8 shows the probability of spectrum utilization *ζ_use_* and the probability of interference to PU *ζ_inf_ vs.* cooperative false alarm probability *Q_f_* with different *η*_1_ and *η*_2_. The performance of the optimal *T_p_* is compared with that of the other *T_p_* in these sub-figures. Figure 8(a,b) reflect spectrum utilization and interference with *η*_1_=1 and *η*_2_=0.1, respectively. The optimal T_p_* = 6.5 s is obtained by solving the optimization problem (17). We can see that the spectrum utilization of the optimal T_p_* = 6.5 s outperforms those of *T_p_* = 4 s, 8 s and 10 s, while the interference of T_p_* = 6.5 s is a little larger than that of *T_p_* = 4 s. That is because if *η*_1_≫ *η*_2_, the loss of spectrum access is reduced greatly. The spectrum utilization and interference with *η*_1_=0.1and *η*_2_=1 are shown in Figure 8(c,d), respectively, and the interference of the optimal T_p_* = 3.2 s is much lower than those of *T_p_* = 4 s, 8 s and 10 s, while the spectrum utilization of T_p_* = 3.2 s is only a little less than that of *T_p_* = 4 s. That is because if *η*_1_≪ *η*_2_, the interference to PU is decreased greatly.

**Figure 9.**
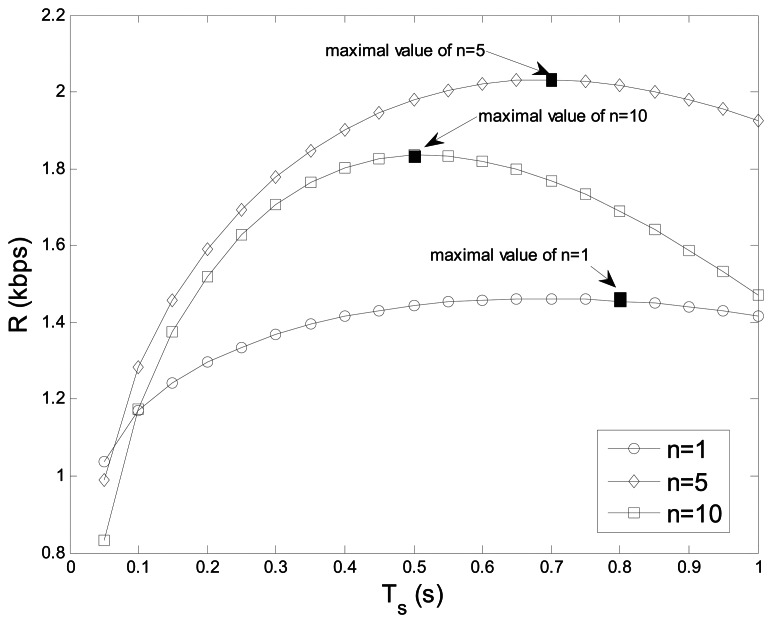
Average throughput *vs.* sensing time.

Figure 9 reflects the average throughput *R vs.* the sensing time *T_s_* with *n* = 1, 5, 10. The convex curves in this figure prove the correctness of the theory proposed in Section 3.2, where exist the maximal values. With the initial increase of *T_s_*, the average throughput improves because of the decrease of the false alarm probability, however, if *T_s_* is larger, the average throughput decreases instead because of the decrease of transmission time. We can also see that the maximal throughput of *n* = 10 is lower than that of *n* = 5 because of the increase of cooperative overhead.

**Figure 10.**
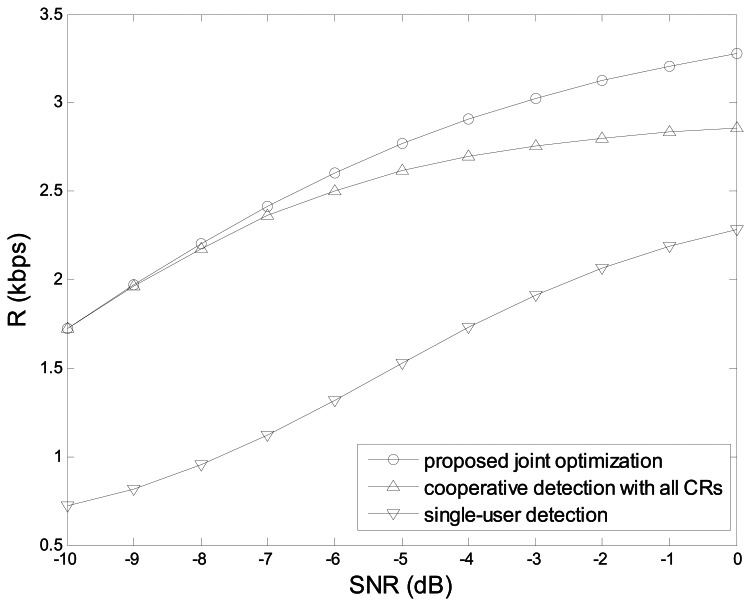
Average throughput *vs.* SNR.

Figure 10 shows the average throughput *_R_ vs.* the average received SNR γ̅ in the three schemes: the proposed joint optimization scheme, the cooperative spectrum sensing with all CRUs, and the single-user detection. We can see that *R* improves with the increase of γ̅, and the proposed optimization scheme outperforms the other two schemes. We also see that if γ̅ is lower, the performances of the proposed scheme and the cooperative spectrum sensing with all CRUs are more approximate. That is because cooperative spectrum sensing needs more collaborative users to decrease the false alarm probability, and therefore the optimal number of CRUs approaches to *N*. However, if γ̅ is larger, the performance of the single-user detection improves much, because the larger number of the collaborative CRUs may increase the cooperative overhead instead of the slight improvement on the detection performance. Hence, the proposed joint optimization scheme is predominant through finding an appropriate number of CRUs.

**Figure 11.**
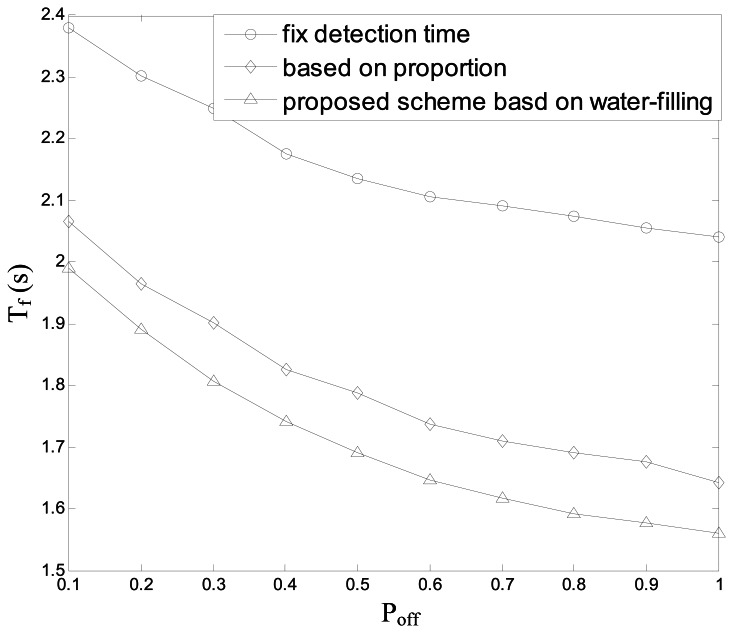
Average searching time *vs.* average idle probability.

Figure 11 indicates the average searching time *T_f_ vs.* the average idle probability of the channels *P_off_* in the three schemes: the searching with the fixed sensing time as Equation (37), the searching with the proportional sensing time as Equation (43) and the proposed scheme based on water-filling principle. In this figure, we can see that the searching time of the proposed scheme decreases observably compared with the other two schemes, because the proposed scheme need not detect the channels with lower *P_off_* in order to save time.

**Figure 12.**
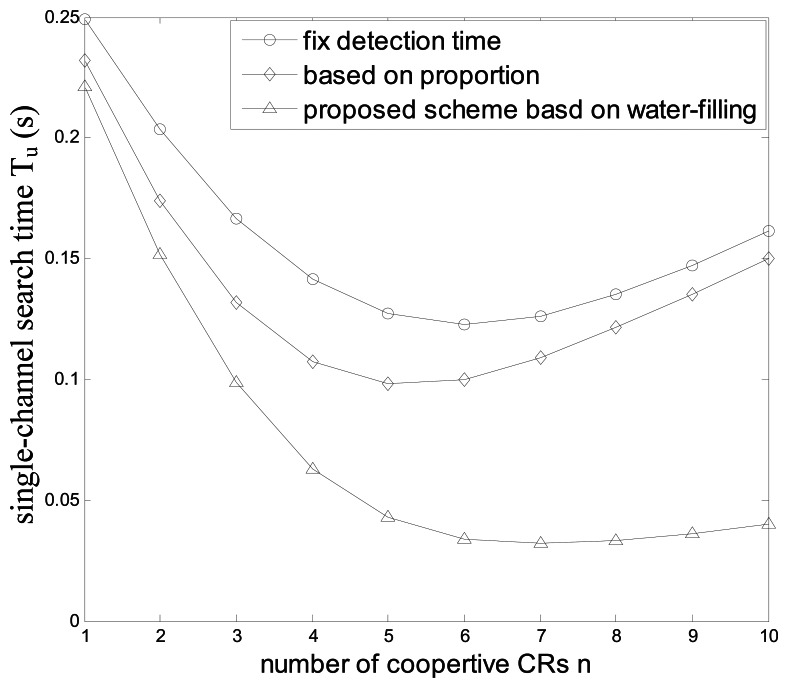
Minimal single-channel searching time *vs.* the number of cooperative CRUs.

Figure 12 compares the minimal single-channel searching time *T_u_* of the three schemes *vs.* the number of cooperative CRUs, and the searching performance of the proposed scheme is always predominant. With the increase of *n*, *T_u_* firstly decreases and then increases, because if *n* is larger, although the sensing performance improves, the increased cooperative overhead may prolong the searching time.

**Figure 13.**
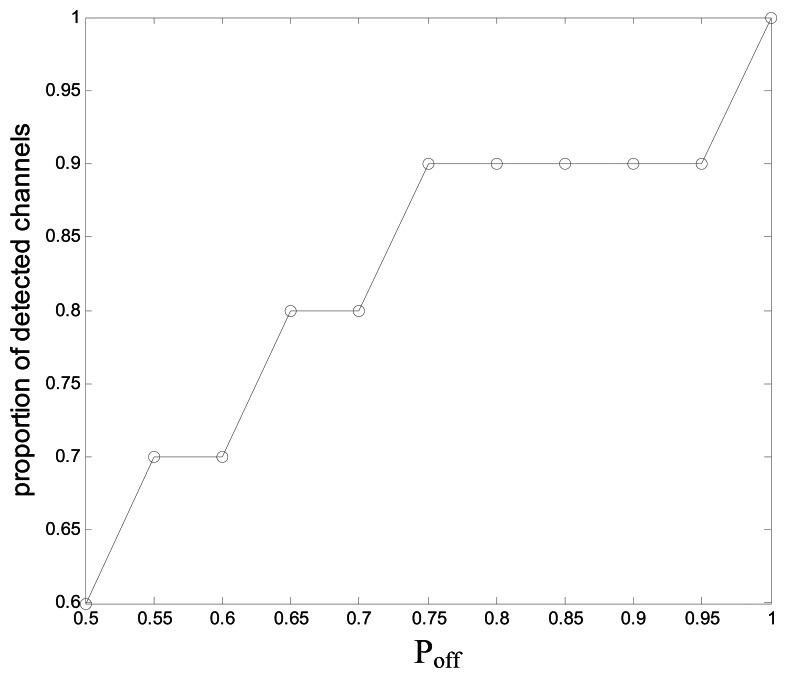
Proportion of detected channels *vs.* average channel idle probability.

Figure 13 shows the proportion of the detected channels to all the *L* channels in the proposed scheme *vs.* the average channel idle probability *P_off_*. With the decrease of *P_off_*, the proportion also declines, and if *P_off_* =0.5, only 60% of channels need to be detected. That is because the number of the channels with lower *P_off_* increases with the decrease of *P_off_*, and the channels with lower *P_off_* should be excluded from the detected channels in order to save the searching time.

## 5. Conclusions

In cognitive radio networks, the interests of PU and CRU are contradictory. In this paper, we consider a cooperative spectrum sensing model where CRU senses the spectrum based on weight fusion periodically in order to avoid interfering PU. The sensing period is firstly optimized for improving the spectrum access and reducing the interference, then the joint optimization algorithm of the sensing time and the number of cooperative CRUs is proposed for making CRU achieve the maximal throughput during each period, and finally the water-filling principle is applied for decreasing the searching time of idle channels. The simulation results show that the significant improvement on the sensing performance and the throughput of CRU has been achieved by the proposed optimization scheme.

## Figures and Tables

**Table 1.** Notation.

**Symbol**	**Denotation**	**Symbol**	**Denotation**
*R*_(*t*)_	received signal	*f_c_*	sampling frequency
*W*	bandwidth of the frequency band	*T*	observed time
*y*_(*t*)_	sampled received signal	*s*_(*t*)_	PU's signal with variance $\sigma_{s}^{2}$
*u*_(*t*)_	Gaussian noise with variance $\sigma_{u}^{2}$	*T*_(*y*)_	statistic of energy sensing
*λ*	sensing threshold	*P_f_*	false alarm probability
*_P_d*	detection probability	*γ*	received signal noise
*_P_m*	miss detection probability	*_T_p*	sensing period
*T_m_*	sensing time	*T_d_*	data transmission time
*T_f_*	Searching time	*N*	number of the CRUs
*L*	number of available channels	*u_j_*	transition rates from busy to idle
*v_j_*	transition rates from idle to busy	$P_{on}^{j}$	busy probability of each channel
$P_{\text{off}}^{j}$	idle probability of each channel	$T_{B}^{j}$	average persistent busy time
$T_{I}^{j}$	average persistent idle time	*_P̅_*_f_	upper limit of false alarm probability
*P̅*_d_	lower limit of detection probability	*T*_loss_	average loss time of spectrum access
*Q_f_*	cooperative false alarm probability	*Q_d_*	cooperative detection probability
*T_inf_*	average interference time	*TS*_loss_	sensing overhead
*Q_loss_*	total sensing loss probability	*T*_m,max_	maximum of sensing time
*η*_1_,*η*_2_	weight factors configured by CR	*ζ*_use_	probability of spectrum utilization
*ζ*_inf_	the probability of interference	*w*	weight vector
*_T_r*	cooperative overhead time	*ξ*	time for sending sensing information
*T_s_*	local detection time	*T_I_*	the average interfering time
*Z*	fusion statistic	*g_i_*	channel gain of CR*i*
*C*_0_,*C*_1_	transmission rates of CR	*R*	the average throughput of CR
*Tu*	sensing time of single channel	$Q_{b}^{j}$	busy probability of channel *j*

## Appendix: Proof of Proposition 1

It can be verified from Equation (35) that the derivative ▽ *Φ*(*T_s_*,*n*) is given by:

(44) $$\begin{array}{ll} \nabla \Phi \left(T_{s},n\right) & =\frac{\partial \Phi \left(T_{s},n\right)}{\partial T_{s}}\\ & =\frac{\beta \left(T_{p}-n\xi -T_{s}\right)}{2\sqrt{2\pi }T_{p}T_{s}}\text{exp}\left(-{\left(\alpha +\sqrt{T_{s}\beta }\right)}^{2}/2\right)-\frac{1}{T_{p}}\left(1-Q\left(\alpha +\sqrt{T_{s}\beta }\right)\right) \end{array}$$

where $\beta =f_{s}\sum_{i=1}^{n}\gamma_{i}^{2}$. Obviously, we have:

(45) $$\{\begin{array}{l} \underset{T_{s}\to T_{p}-n\xi }{\lim}\nabla \Phi \left(T_{s},n\right)<-\frac{1}{T_{p}}\left(1-Q\left(\alpha \right)\right)<0\\ \underset{T_{s}\to 0}{\lim}\nabla \Phi \left(T_{s},n\right)=+\infty \end{array}$$

Equation (45) means that *Φ*(*T_s_*,*n*) is a increasing function if *T_s_* approaches to zero and a decreasing function if *T_s_* approaches to *T_p_* − *nξ*. Hence, there is a maximal point of *T_s_* within the interval [0, *T_p_*−*nξ*].
